# Efgartigimod treatment for therapy-refractory autoimmune encephalitis with coexistent NMDAR and LGI1 antibodies: a case report and literature review

**DOI:** 10.1007/s10072-024-07843-8

**Published:** 2025-01-17

**Authors:** Jiaming Xu, Zhenli Guo, Jie Zhao, Yun Chen, Zhijun Liu, Yan Wu

**Affiliations:** 1 Department of Neurology, Union Hospital, Tongji Medical College, Huazhong University of Science and Technology, Wuhan, China; 2 Department of Neurology, Hubei Provincial Hospital of Integrated Chinese and Western Medicine, Xinhua Hospital of Hubei University of Chinese and Western Medicine, Wuhan, China; 3 Department of Radiology, Union Hospital, Tongji Medical College, Huazhong University of Science and Technology, Wuhan, China

**Keywords:** Autoimmune encephalitis, N-methyl-D-aspartate receptor, Anti-leucine-rich glioma-inactivated 1, Efgartigimod, Treatment

## Abstract

The Fc receptor (FcRn) inhibitors can ameliorate autoimmune conditions such as myasthenia gravis through a rapid and specific clearance of serum IgG levels, and they also have potential for future use in a wider variety of antibody-mediated autoimmune diseases. Some patients with therapy-refractory autoimmune encephalitis (AE) continue to be unresponsive to initial and secondary treatment regimens. A 32-year-old male presented with predominant psychiatric symptoms and seizures, along with imaging evidence indicating multifocal cerebral cortical involvement. Neural antibody testing revealed dual positivity for N-methyl-D-aspartate receptor (NMDAR) and leucine-rich glioma-inactivated 1 (LGI1) antibodies in both serum and cerebrospinal fluid (CSF). Human leukocyte antigen (HLA) genotyping revealed the presence of the DQB1*03:01 and DQB1*06:01 alleles in the patient. Treatment with efgartigimod, the FcRn inhibitor, led to significant clinical improvements accompanied by a significant decrease in both anti-NMDAR and anti-LGI1 antibody levels. Herein, we report a rare case of therapy-refractory anti-NMDAR AE coexisting with positive LGI1 antibodies. Efgartigimod demonstrates promising potential for treating antibody-mediated AE.

**Clinical trial number** Not applicable.

## Introduction

Antibody-mediated autoimmune encephalitis (AE) is a multifaceted neurological condition characterized by brain inflammation resulting from an autoimmune response, where autoantibodies target synaptic receptors on neuronal or glial cell surfaces. The presence of specific antibodies targeting antigens within the central nervous system contributes to the pathogenesis of AE. Antibodies against various antigens, including the N-methyl-D-aspartate receptor (NMDAR), leucine-rich glioma-inactivated 1 (LGI1), and contactin-associated protein-like 2 (CASPR2), have been detected in individuals with AE [1–3]. These antibodies correlate with distinct clinical presentations and prognostic outcomes among AE patients [4]. NMDAR antibodies are linked to symptoms such as acute psychosis, memory disturbances, and seizures, primarily occurring in young women with ovarian teratoma. On the other hand, LGI1 antibodies are typically detected in older patients and are associated with symptoms affecting the limbic system, often without associated tumors. Cerebrospinal fluid (CSF) findings in AE associated with NMDAR and LGI1 antibodies exhibit disparities, with anti-NMDAR AE demonstrating more pronounced inflammation [5]. In the context of therapy-refractory AE with coexistent NMDAR and LGI1 antibodies, the management of these patients poses a significant challenge. Treatment options for AE often involve immunosuppression therapy, such as corticosteroids and immunosuppressive agents. Responses to corticosteroid therapy in instances of AE with concurrent NMDAR and LGI1 antibodies can vary, as demonstrated by a case wherein symptoms deteriorated upon dosage reduction [6]. Studies have demonstrated the efficacy of combining immunoadsorption therapy with immunosuppression in patients with AE, particularly those harboring surface antibodies such as LGI1 and CASRP2 [1]. Additionally, therapeutic plasma exchange has been recommended as a first-line therapy for conditions like anti-NMDAR AE [6]. Studies have shown that early treatment of certain AE with rituximab or cyclophosphamide improves outcome when corticosteroids, intravenous immunoglobulin (IVIG), and plasma exchange have proven ineffective [7]. The clinical characteristics and short-term prognosis of AE can also differ based on the specific antibody present in the plasma or CSF. For example, patients with anti-NMDAR encephalitis may experience acute psychosis and respiratory dysfunction, and exhibit decreased inflammatory cytokine production of antigen-specific CD4 + T cells, while those with anti-LGI1 encephalitis may exhibit symptoms related to the limbic system, highlighting the importance of understanding the immune response in these patients [8]. Furthermore, the economic burden of patients with AE, including those with anti-NMDAR, anti-γ-aminobutyric acid-B receptor (GABAR), anti-LGI1, and anti-CASPR2 antibodies, has been evaluated in Western China. The direct costs associated with anti-LGI1/CASPR2 encephalitis were notably lower than those linked with anti-NMDAR encephalitis and anti-GABAR encephalitis [9]. This data emphasizes the necessity for implementing cost-effective management approaches for individuals with therapy-resistant AE. Nevertheless, a few AE patients continue to be refractory to initial and secondary treatment modalities, necessitating the development of more efficacious escalation strategies [8]. Recently, efgartigimod has been approved for treatment of generalized myasthenia gravis in adults [9, 10], directly targeting the neonatal Fc receptor (FcRn) and selectively reducing serum IgG concentration [11]. This therapy has shown promise in ameliorating autoimmune conditions such as myasthenia gravis [12]. By accelerating the destruction of IgG, efgartigimod reduces pathogenic IgG and IgG immune complexes [13]. Efgartigimod treatment results in a rapid and specific clearance of serum IgG levels, warranting further evaluation in IgG-driven autoimmune diseases [14]. Clinical trials have demonstrated the safety and efficacy of efgartigimod in treating IgG-driven autoimmune diseases [15]. The evolution of anti-B cell therapeutics in autoimmune neurological disorders has also highlighted the role of efgartigimod in managing conditions like myasthenia gravis [16]. Efgartigimod has also being studied in adults with other IgG antibody-mediated neurological diseases, including chronic inflammatory demyelinating polyneuropathy and stiff-person syndrome, and often shows a great potential to treat these diseases [17]. In conclusion, the coexistence of NMDAR and LGI1 antibodies in patients with therapy-resistant AE poses a distinctive clinical challenge. Gaining a comprehensive understanding of the link between the specific antibody profiles and treatment responses is crucial for optimizing the management of these patients. Additional research is warranted to elucidate the underlying pathophysiology and develop targeted therapeutic strategies for patients with AE. Here, we describe the case of a 32-year-old man suffering from a severe refractory course of AE with coexistent NMDAR and LGI1 antibodies, with significant clinical improvements after the use of efgartigimod.

## Article types

Review.

## Manuscript formatting

### Case report

**Fig. 1:**
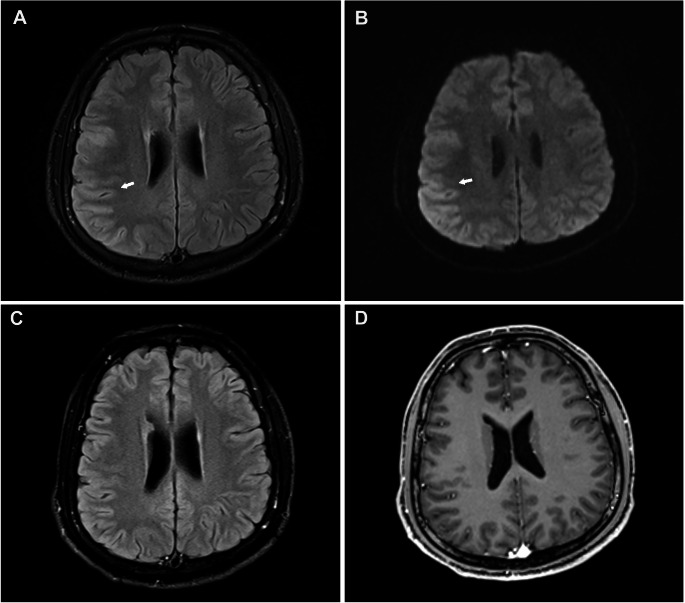
Brain magnetic resonance imaging. **A** and **B** Brain MRI shows T2/FLAIR and DWI signal hyperintensities involving multiple cortical areas of the right temporal, occipital and parietal lobes (arrow). **C** and **D** T2/FLAIR and contrast-enhanced MRI four months after symptom onset showed no specific abnormalities in the cerebral cortex

**Fig. 2:**
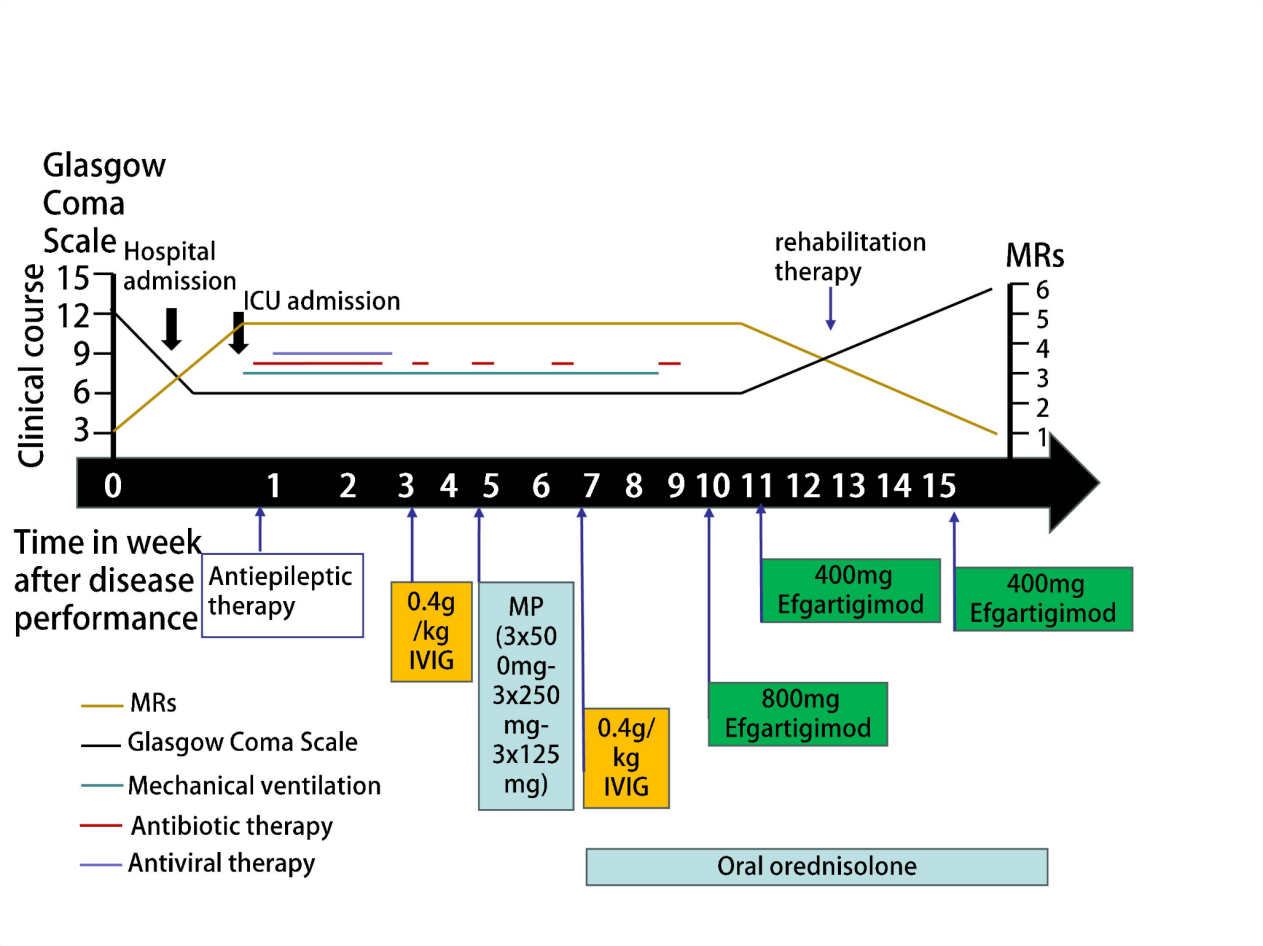
Overview of patient’s disease history. Patient’s clinical course after initiation of immunotherapy. The graphic illustration depicts patient’s course of modified Rankin Scale, duration of mechanical ventilation, as well as the history of infections with administered antibiotic and antiviral therapies

**Fig. 3:**
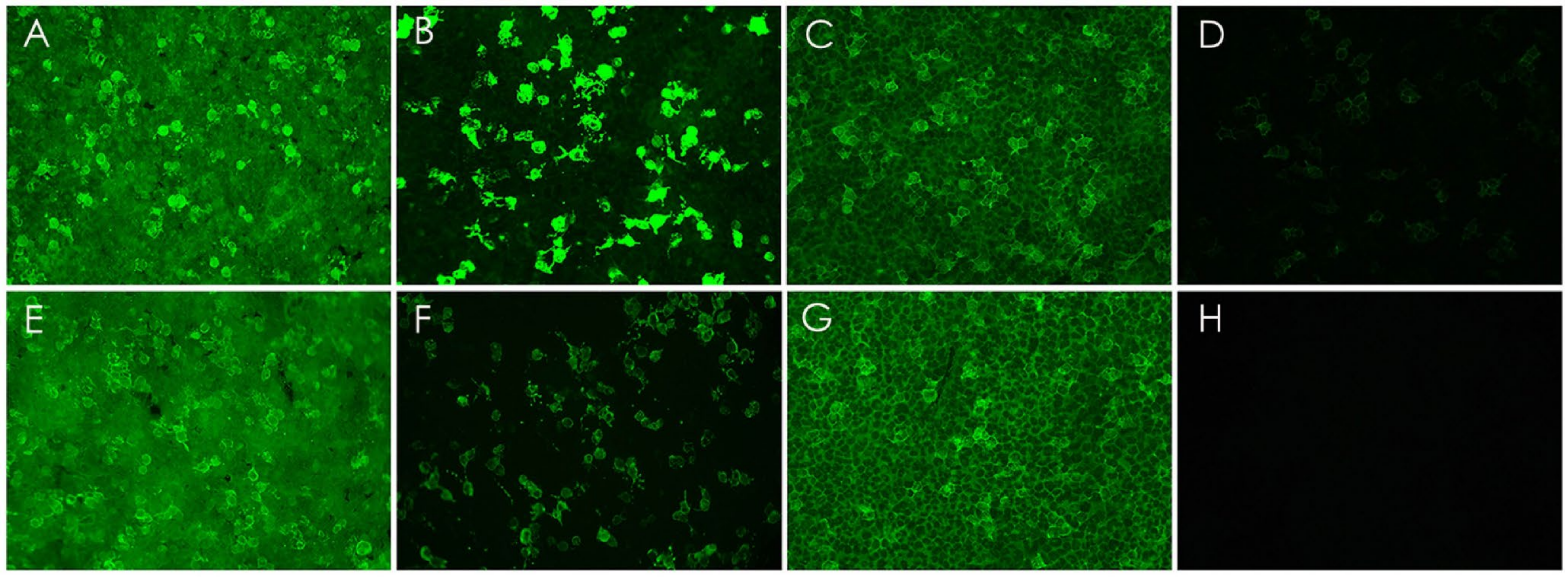
N-methyl-D-aspartate receptor (NMDAR) and leucine-rich glioma-inactivated 1 (LGI1) antibodies in serum and CSF validated by cell-based assays. NMDAR-IgG levels in serum (A, 1:100) and CSF (B, 1:32) at disease onset, and in serum (E, 1:32) and CSF (F, 1:10) over two weeks after efgartigimod treatment initiation. LGI1-IgG levels in serum (C, 1:10) and CSF (D, 1:1) at disease onset, and in serum (G, 1:10) and CSF (H, negative) over two weeks after efgartigimod treatment initiation

A 32-year-old man was admitted to the emergency unit of Union Hospital, Tongji Medical College, Huazhong University of Science and Technology, Wuhan, China, due to behavioral changes and psychiatric symptoms persisting for 48 h. The patient was healthy before and with the absence of personal or family history of genetic, autoimmune, and neurological disorders. On physical examination, the patient was unconscious with a Glasgow Coma Scale (GCS) of 8(E2 + V3 + M3). Subsequently, the patient experienced generalized tonic–clonic seizures in the emergency unit. Neurological examination showed prominent stiff neck, confusion of consciousness, sluggish pupillary light responses, and increased muscle tension. No autonomic or sleep disturbances were noted. With the rapid deterioration of the clinical condition, brain magnetic resonance imaging (MRI) was conducted, revealing T2/FLAIR and DWI signal abnormalities in multiple cortical areas of the right temporal, occipital, and parietal lobes (Fig. 1). Then, he developed severe lung infections and acute respiratory failure during hospitalization, and was immediately transferred to the intensive care unit (ICU). Subsequently, he underwent intubation and tracheotomy for prolonged ventilator support. Further details of disease course are summarized in Fig. 2. Treatment initiation focuses on controlling the lung infection and alleviating symptoms of respiratory failure. The high-dose cefoperazone-sulbactam combined with tigecycline have been selected as therapeutic options for respiratory infections caused by pseudomonas aeruginosa isolated from bronchoalveolar lavage fluid. Initially, he experienced seizures approximately once every three days during hospitalization. His seizure was well controlled with the combination of levetiracetam and oxcarbazepine. Furthermore, next-generation sequencing (NGS) testing detected Epstein-Barr virus (EBV) positivity in the CSF, prompting the prescription of aciclovir antiviral medication. Electroencephalogram (EEG) showed that in a state of coma, the background activity is predominantly characterized by widespread moderate to low amplitude slow waves or rhythmic sharp wave activity around 2 Hz, occasionally lateralized to the left. No distinct sleep-wake cycles were observed during 15 h of monitoring. Computed tomography (CT) scan of chest and abdomen was unremarkable for malignancy. The CSF was colorless and transparent, with a pressure of 120 mmH2O. Routine biochemistry revealed leucocytes 6 × 10 [6]/L, protein 0.42 g/L, glucose 3.8 mmol/L, and chloride 124.0 mmol/L. Antibodies related to paraneoplastic processes were not detected in the blood and CSF. Specifically, the anti-Hu, Yo, Ri, Ma2, CV2, and amphiphysin antibodies were all negative. The anti-NMDAR antibody titers were positive in CSF (1:100) as well as serum (1:32). In the meantime, the anti-LGI1 antibodies in both serum (titer of 1:10) and CSF (titer of 1:1) were also detected by fixed cell-based assay (Fig. 3). However, despite two months of combined steroid/IVIG immunotherapy initiated according to the Chinese Expert Consensus on the Diagnosis and Treatment of Autoimmune Encephalitis (2022 Edition) following 2 weeks of antibiotic treatment, which included a cycle of methylprednisolone (0.5 g/day to 0.125 g per day for 3 days, followed by an oral prednisone 1 mg/kg/day and the patient’s weight is 79 kg) and two cycles of IVIG treatments (0.4 g/kg/day for 5 consecutive days), the patient’s clinical state has not improved. Repetitive testing of CSF and serum NMDAR and LGI1 antibodies remained unchanged. Since there was no clinical improvement 10 weeks after onset, three times of efgartigimod (800 mg on day 1, 400 mg on days 7 and 37) were administered intravenously. His clinical signs, especially psychiatric symptoms, were significantly improved within 7–10 days of starting efgartigimod therapy. During the last follow-up, 1 month after the second efgartigimod treatment, the patient gradually regained consciousness, and regained the ability to communicate verbally and walk without assistance following rehabilitation training with hyperbaric oxygen therapy, with an mRs score of 1 and a GCS score of 14(E4 + V4 + M6). Repeat examination of antibodies showed decreased anti-NMDAR antibodies both in CSF (titer of 1:32) and serum (titer of 1:10), and reduced anti-LGI1 antibodies in serum (titer of 1:10) but the paired CSF is negative (Fig. 3). A follow-up MRI performed four months after the onset showed no specific abnormalities in the cerebral cortex (Fig. 1). Human leukocyte antigen (HLA) genotyping showed the patient carried the DQB1*03:01 and DQB1*06:01 alleles.

## Discussion

This study represents the first report of efgartigimod use in a treatment-refractory case of AE mediated by dual antibodies. Our findings demonstrate that efgartigimod, when combined with other concurrent immunotherapies, leads to significant clinical improvements as well as reductions in antibody levels.

While the occurrence of multiple anti-neuronal antibodies in AE patients is rare, cases of AE with the coexistence of double or more anti-neuronal antibodies have been documented in a few case series or retrospective studies [18–21]. AE-associated antibodies are typically classified into two groups: those targeting specific neuronal surface receptors (e.g. anti-NMDAR or γ-aminobutyric acid-B receptor (GABABR), anti-LGI1 or CASPR2, anti-DPPX or IgLON5) and those targeting intracellular neuronal antigens (e.g. anti-Hu or Yo, anti-Ri or SOX1, anti-GAD or anti-CV2) [18, 22]. NMDARs are localized in neuronal post-synaptic membranes of the neuromuscular junction with crucial roles in synaptic transmission and plasticity [23]. LGl1 is a secreted neuronal protein localized at both pre- and postsynaptic membranes, that interacts with the ADAM (disintegrin and metalloproteinase domain-containing protein, ADAM) protein complex, essential for neuronal excitability and synaptic transmission by regulating the expression of AMPA (α-amino-3-hydroxy-5-methyl-4-isoxazolepropionic acid, AMPA) receptors [24]. Various antibodies have been identified in association with AE, each of which can result in different symptoms and outcomes [25]. The classic presentation anti-NMDAR AE has been described as an acute to subacute onset psychosis and seizures [26]. Anti-LGI1 AE is characterized by rapid progressive dementia, sleep disorders, refractory hyponatremia, and fascio-brachial dystonic seizures [27, 28]. The classic presentation of anti-CASPR2 antibodies has been described as limbic encephalitis, peripheral nerve hyperexcitability often with or without symptoms indicative of Morvan syndrome, neuropathic pain, and manifestations affecting the cerebral or cerebellar regions [29]. Anti-AMPA receptor encephalitis presents with the typical symptoms of rapidly progressive limbic encephalitis and is typically associated with thymic tumors [30]. We provide a summary of the frequency of detection and clinical characteristics of anti-LGI1, anti-NMDAR, anti-CASPER2, and anti-AMPAR in Table 1.

**Table 1:** Summarize the characteristics of clinical and outcomes in anti-LGI1, anti-NMDAR, anti-CASPER2, and anti-AMPAR autoimmune encephalitis

Neuronal antibody	NMDAR	AMPAR	LGI1	CASPR2
Frequency	36%	9%	9%	9%
Characteristics of the patient cohort	Children and young adults, predominantly females with median age 21	Middle-aged females	Elders, Median age 60 years	Elders, Median age 60 years
Tumor	Ovarian teratoma	Thymomas	Rare	Thymomas
Pathogenesis	Neoplastic lesions and virus-infected neurons	Specific tumor antigens	Participating in the formation of pre- and post-synaptic protein complexes	Participating in the formation of pre- and post-synaptic protein complexes
Clinical presentation	Psychotic symptoms	Limbic encephalitis, cognitive changes, psychiatric disorders, epilepsy	FBDS, limbic encephalitis with seizures, memory deficits, confusion and behavioral changes	Epileptic seizures, memory deficits, mental disorders, and disturbances of consciousness
Clinically characterized	Acute	Acute or subacute	Gradual	Subacute
Treatment	Tumor resection and immunotherapy	Immunotherapy and thymoma therapy	Immunotherapy	Immunotherapy
Outcomes	Good outcome	Good outcome or partial response	Good outcome	Good outcome
CSF	Inflammatory changes	Inflammatory changes	Normal or inflammatory changes	Normal or inflammatory changes

Abbreviations: NMDAR: N-methyl-D-aspartate receptors; AMPAR: α-amino-3-hydroxy-5-methyl-4-isoxazole propionate receptors; CASPR2: Contactin associated protein 2; LGI1: Leucine-rich Glioma Inactivated 1; CSF, cerebrospinal fluid; FBDS, Faciobrachial Dystonic Seizures

The co-occurrence of multiple autoantibodies is a rare phenomenon, research has indicated that less than 10% of AE cases involve multiple anti-neuronal antibodies [18]. In a multicenter retrospective study on the coexistence of multiple anti-neuronal antibodies in Chinese patients with AE, the researchers have found that anti-NMDAR and anti-GABABR antibodies were the two most common anti-neuronal surface antigen antibodies in AE patients with the coexistence of multiple anti-neuronal antibodies [18]. To our knowledge, there have only been five reported AE cases with co-occurring NMDAR and LGI1 antibodies [18, 31–33]. Although the underlying mechanism behind this poly-immunoreactivity remains unclear, the coexistence of multiple antibodies may be partially explained by the concept of epitope spreading, in which persistent recognition of self-antigens leads to chronic activation of the immune system [34]. The Table 2 includes a summary for the clinical features, treatment options, and outcomes in three primary types of combination of double antibodies: anti-LGI1 and anti-CASPR2, anti-NMDAR and anti-CASPR2, and anti-NMDAR and anti-LGI1. Patients with mono-antibody AE often exhibit symptoms specific to the targeted antigen. The prognosis in mono-antibody AE can vary, influenced by factors such as prompt diagnosis, initiation of appropriate treatment, and the severity of neurological involvement. However, patients with multiple antibodies may present a more heterogeneous clinical picture, reflecting the combination of antigens involved. The clinical presentation may vary based on the predominant antibody and the interactions between the two antibodies. This interaction can lead to a complex clinical phenotype. Early recognition and treatment often result in significant symptom improvement and functional recovery. This complexity poses challenges in diagnosis and management, and the presence of multiple antibodies may complicate treatment response and prognosis. Treatment strategies for dual antibody-mediated AE typically involve a combination of immunotherapy, such as corticosteroids, IVIG, plasma exchange, and immunosuppressive agents like rituximab and cyclophosphamide. The choice of treatment may be guided by the predominant clinical features and the severity of neurological symptoms. Optimal therapeutic regimens for dual antibody AE are still being explored, and personalized treatment approaches may be necessary based on individual patient characteristics [32, 35–40]. Notably, the refractory hyponatremia is a characteristic feature of anti-LGI1 AE, and 60%~ 88% of the patients with anti-LGI1 antibodies may develop hyponatremia during the course of the disease [41, 42]. In our patient, an acute onset psychosis and seizures was observed, and there is no evidence of hyponatremia in the affected individual. Therefore, the clinical picture of our patient was dominated by features of anti-NMDAR encephalitis, as reflected by the results of the higher anti-NMDAR antibodies titers than anti-LGI1 antibodies. In addition, pathogen analysis revealed a higher specificity of CSF EBV DNA in our case, which may play a critical role in driving immunopathology in antibody-mediated AE.

**Table 2:** Summary for the clinical features, treatment options, and outcomes in three primary types of combination of double antibodies: anti-LGI1 and anti-CASPR2, anti-NMDAR and anti-CASPR2, and anti-NMDAR and anti-LGI1

Antibody combinations	Reports	Clinical Presentation	Treatment	Tumor	Outcome
**NMDAR and CASPR2**	Qiao S et al. [37]	Headache, seizures, numbness	GC, IVIG	No	Improvement
	Qiao S et al. [37]	Fever, headache, impaired memory, seizures, impaired consciousness, coma	GC, IVIG	No	Improvement
	Qiao S et al. [37]	Fever, diarrhea, headache, memory loss, paroxysmal involuntary movements and pain in both lower limbs	GC, IVIG	No	Improvement
	Zhou Y et al. [40]	Seizures, sleep disorder and cognitive impairment	Anticonvulsant therapy, GC	No	Sporadic epilepsy
	Zhou Y et al. [40]	Insomnia, a speech disorder, emotional lability, and confusion	Acyclovir	No	Fully recovered
	Zhou Y et al. [40]	Depression, severe insomnia, visual and acoustic hallucinations, and a suicide attempt	Plasmapheresis, GC IVIG and mycophenolate mofetil	No	Improvement
**NMDAR and LGI1**	Wang XJ et al. [32]	Seizures, memory loss, dystonia, drowsiness	GC, IVIG	Lung cancer	Improvement
	Wang XJ et al. [32]	Seizures, memory loss, psychosis, hyponatremia	GC	No	Improvement
	Qiao S et al. [37]	Memory impairment, seizures, FBDS	GC, IVIG	No	Improvement
	Liu XY et al. [34]	Seizure, consciousness disturbance	GC, IVIG	No	Improvement
	Ji T et al. [33]	Seizures, behavioral changes, psychosis, sleep disorders, decreased consciousness, and FBDS	GC, Acyclovir	No	Improvement
	Xu JM et al. [56]	Seizures, stiff neck, confusion of consciousness, sluggish pupillary light responses, and increased muscle tension	GC, IVIG, Efgartigimod treatment	No	Improvement
**LGI1 and CASPR2**	Zhou Y et al. [40]	Memory decline, a speech disorder, and confusion	GC, IVIG	No	Improvement
	Ren HT et al. [38]	Seizure, apnea, myalgia, fibrillation	GC, IVIG	No	Improvement
	Qi HC [36]	Seizures, memory impairments	GC, IVIG	No	Improvement
	Qiu ZD et al. [39]	Weakness, cognitive impairment	IVIG	Thymoma	Improvement
	T. Yeo et al. [41]	Complex partial seizures, limbic encephalitis	Immunotherapy	Yes	Improvement

Abbreviations: NMDAR: N-methyl-D-aspartate receptors; CASPR2: Contactin associated protein 2; LGI1: Leucine-rich Glioma Inactivated 1; GC: glucocorticoids; IVIG: intravenous human immunoglobulin

Existing treatment options for therapy-refractory AE are quite limited, especially for those with coexistence of multiple anti-neuronal antibodies. Earlier initiation of immunotherapy (IVIG or prednisolone) and escalation to second-line therapy in those with therapy-refractory AE may improve patient outcomes. If patients show no clinical improvement or only minimal improvement 2 weeks after initiating first-line treatment, it is recommended to proceed with second-line therapies [43, 44]. Current treatment for AE is based on immunotherapy, with an escalating approach starting with steroids, immunoglobulins, and/or plasma exchange. If first-line therapy fails, second-line immunotherapy such as rituximab is typically employed. Efgartigimod, along with other monoclonal antibodies such as tocilizumab, is being investigated as a potential third-line therapy for AE [9]. Studies have shown that tocilizumab, an anti-interleukin-6 antibody, may be effective in treating AE that is refractory to conventional immunotherapies and rituximab [45]. Research suggests that efgartigimod and other newer monoclonal antibodies could play a role in the future treatment of AE [9]. In our case, after 8 weeks of treatment with IVIG and prednisolone, the patient’s clinical manifestation and ancillary investigations did not show any significant improvement. Considering the patient’s current severe pulmonary infection, the fact that infections (all of them pneumonitis) accounted for 11.3% of the adverse events of rituximab treatment [9], and the potential reduction in vaccination response during inflammatory pulmonary infections, we opted for efgartigimod as a second-line therapy. Since efgartigimod is not a standard second-line treatment for AE, its administration involves communication with the family and obtaining their consent. This refractory case showed improvement after 1 week of efgartigimod treatment. However, the long-term clinical outcome of efgartigimod in patients with therapy-refractory AE, especially those with double or more anti-neuronal antibodies, remains a concern.

A study conducted on participants with pemphigus revealed that efgartigimod resulted in a reduction in total IgG levels, autoreactive IgG antibody levels, and desmoglein-specific B cells, which are directly involved in pemphigus pathology [46]. Interestingly, although total IgG levels returned to baseline after cessation of efgartigimod treatment, autoreactive antibody levels remained low in some participants, indicating a sustained reduction in pathogenic IgG levels. Additionally, efgartigimod had no effect on total leukocytes, neutrophils, monocytes, or lymphocytes in patients undergoing extended therapy. These findings suggest that efgartigimod exerts an immunomodulatory effect beyond IgG recycling blockade, contributing to improved clinical conditions in pemphigus patients. Further studies are required to validate these observations and establish long-term clinical responses in autoimmune diseases [47]. In another study focusing on patients with generalized myasthenia gravis, efgartigimod demonstrated efficacy and tolerability in improving symptoms. Patients treated with efgartigimod exhibited a higher proportion of Myasthenia Gravis Activities of Daily Living (MG-ADL) responders compared to those receiving a placebo, indicating a positive treatment response. The safety profile of efgartigimod was favorable, with headache and nasopharyngitis being the most common adverse events [48]. The Table 3 summarizes the use of efgartigimod in the treatment of conditions other than myasthenia gravis, for example bullous pemphigoid, chronic inflammatory demyelinating polyradiculoneuropathy, immune thrombocytopenia, myasthenia gravis, autoimmune myositis and pemphigus. The study underscored the individualized dosing based on clinical response as a distinctive feature of efgartigimod treatment.

**Table 3:** Summary for the use of efgartigimod in the treatment of conditions other than myasthenia gravis

Reports	Diseases	Outcome
Broome C. and Newland AC et al. [57–59]	Primary immune thrombocytopenia	Rapid increasing in platelet counts, effectively reducing total IgG levels
Goebeler M et al. and Maho-Vaillant M et al. and Zakrzewicz A et al. [48, 59, 60]	Pemphigus vulgaris and foliaceus, pemphigus.	Early disease control and complete clinical remission
Reznik SE et al. [61]	Post COVID-19 syndrome	Improving the quality of life
Allen J et al. [62]	Chronic Inflammatory Demyelinating Polyneuropathy	Reducing the risk of clinical deterioration
Di Stefano V et al. [63]	Stiff-person syndrome	Effective in reducing the overall burden of SPS

The HLA-B*07:02 [49] and HLA-DRB1*16:0.2 [50] have been reported to be associated with increased susceptibility to anti-NMDAR encephalitis. Anti-LGI1 encephalitis is strongly associated with the DRB1*07:01-DQB1*02:02 haplotype, as well as with the DRB1*03:01 allele [51, 52]. These suggest that there may be a genetic basis for susceptibility of the development of antibodies-mediated encephalitis. Hu et al. compared and analyzed the HLA genotypes of 101 patients with AE (77 anti-NMDAR, 11 anti-LGI1, and 13 anti-GABAR antibody-positive patients, respectively) to those of 200 healthy controls. The results of the study indicated that DRB1*03:01 or DQB1*02:01 allele and the extended DRB1*03:01 ~ DQB1*02:01 haplotype represented strong susceptibility loci for anti-LGI1 encephalitis. Additionally, the DRB1*08:03 ~ DQB1*06:01 or B*08:01 ~ C*07:02 haplotype was likely to be associated with anti-LGI1 encephalitis [53]. In our study, HLA analysis showed that the patient carried the HLA-DQB1*03:01/06:01 alleles, carried the alleles (HLA- DQB1*06:01) which may contribute to AE susceptibility. It has been reported that the three patients with co-existent CASPR2 and LGI1 antibodies, carried HLA-B*44:02, HLA-C*05:01, HLA-DQA1*03:01 and HLA-DQB1*03:01, representing a different complement of alleles compared to patients with antibodies to either LGI1 or CASPR2 [54]. Thus, the HLA-DQB1*03:01/06:01 alleles identified in the patient might be associated with susceptibility of both encephalitis, reflecting that the specific combination of anti-neuronal antibodies.

Nevertheless, there are some limitations should be addressed. Firstly, we did not take advantage of flow cytometry to evaluate the impact of efgartigimod on the T and B lymphocytes response before and after efgartigimod treatment, and immunoglobulin tests were not conducted post-treatment. Another limitation is that the efgartigimod treatment results in marked clinical improvements, which depends in part on prior methylprednisolone/intravenous immunoglobulin though with little clinical benefit. Furthermore, the clinical observation has been made just in a single case, the effect of efgartigimod treatment in patients of therapy-refractory AE mediated by multiple antibodies require a large sample size. Finally, antibodies-mediated AE may be associated with underlying malignancy, and thus the co-existent of multiple auto-antibodies raises the probability of an underlying malignancy. Neoplasm cannot be completely ruled out in the patient, although there was no significant disclosure during a CT scan. The PET/CT for malignancy screening was not performed in the patient due to an exacerbation of his respiratory status, and although testing was suggested after discharge, the patient’s malignancy screening results were not available during the late follow-up.

In conclusion, we describe a unique case of anti-NMDAR encephalitis with co-occurring LGI1 antibodies presenting with predominant psychosis and seizures symptoms. Our results suggest that efgartigimod can serve as a potent therapeutic agent in severe double antibody-medicated AE, but its clinical efficacy and safety requires to be evaluated in further prospective studies with larger sample sizes. It is noted that the co-existence of multiple anti-neuronal antibodies might cause the overlapping of clinical features, and mediate adverse pathogeneses of patients with AE. Long-term efficacy and safety of potential therapeutic applications for multiple antibody-medicated AE should be evaluated comprehensively.
